# Sarcoidosis And Atrial Fibrillation: A Rare Association And Interlink With Inflammation

**DOI:** 10.1016/s0972-6292(16)30569-1

**Published:** 2012-12-02

**Authors:** Uma N Srivatsa, Jason Rogers

**Affiliations:** UC Davis Medical Center, 4860 Y street, Ste 2820, Sacramento, CA

**Keywords:** atrial fibrillation, sarcoidosis, inflammation

## Abstract

We report a case of sarcoidosis presenting initially as atrial fibrillation(AF). His response to anti-arrhythmic treatment strategy was suboptimal. On initiation of immunosuppressive therapy, AF was better controlled. This interesting case highlights a likely link between inflammation and pathogenesis of atrial fibrillation.

Atrial fibrillation (AF) has been associated with acute inflammation, post operative states and intensive care settings, with documented higher levels of inflammatory markers. Cases of left atrial tachycardia with sarcoidosis [1], a chronic inflammatory state have been reported, however a direct relation of AF to inflammation in this condition has not been shown. We would like to submit a case with sarcoidosis, whose intial presentation was an episode of AF.

A 39 yr old Caucasian male presented with paroxysmal AF 2 years ago. After a failed trial of anti-arrhythmic agents, he underwent circumferential ostial and segmental pulmonary vein isolation. However, AF recurred within six months, and subsequently he also had an episode of syncope. Since evaluation with 48-hour Holter monitor revealed episodes of sinus arrest, he was implanted a dual chamber permanent pacemaker. He was again treated with class III anti-arrhythmic agents with no reduction in episodes of AF. During this presentation, his chest radiography revealed lung infiltrates, and ECG showed prolonged PR interval, a rate related RBBB with left posterior hemiblock (trifascicular block) which later on persisted with sinus rhythm in the physiologic heart rate range. (Figure 1). He underwent computerized tomography of chest revealing multiple systemic lesions, the biopsy of which revealed granulomas. A diagnosis of sarcoidosis was made and he was started on prednisone, which was noted subsequently to reduce AF burden on pacemaker interrogation. Since he showed clinical improvement, hydroxychloroquine was introduced as a steroid sparing agent with consequent further reduction in AF burden. (Figure 2) Withdrawal of prednisone (phase 4 in Figure 2) led to a slight increase in AF burden. The improvement in AF burden paralleled that in lungs as noted in serial CT scans of chest. One year after the diagnosis of sarcoidosis, he developed cardiomyopathy and had non-sustained ventricular tachycardia. A Gallium scan confirmed two areas of uptake in the left ventricle - anteroapical septum and basal septum confirming sarcoidosis of the heart. In view of these findings, his pacemaker was upgraded to implantable cardioverter defibrillator. Also, his drug regimen has been changed to mycophenolate 1000 mg twice daily. This is a unique patient, where the presenting cardiac symptoms were that of AF and subsequently conduction system disease, ventricular tachycardia and cardiomyopathy due to sarcoidosis were seen. Also, improvement in AF burden paralleling clinical and pulmonary improvement was shown with anti-inflammatory drug management.

Steroids and steroid sparing agents have been used to treat cardiac sarcoidosis.[2] While AV conduction blocks, ventricular tachycardia and cardiomyopathy are known associations, [3] AF is unusual secondary to sarcoidosis. Improvement in AF burden by anti-inflammatory agents in cardiac sarcoidosis has been shown for the first time in literature.

## Figures and Tables

**Figure 1:**
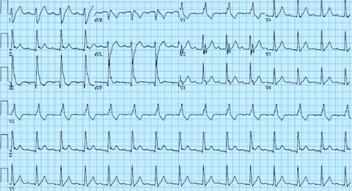
Twelve lead EKG revealing prolonged PR interval, left posterior hemiblock and RBBB

**Figure 2:**
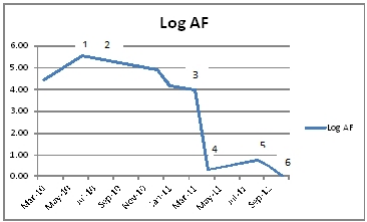
Log of AF duration over the course of anti-inflammatory treatment 1-Prednisone 20 mg once daily 2-Prednisone 5 mg once daily, hydroxychloroquine200 mg twice daily 3-Prednisone 40 mg every other day and hydroxychloroquine 200 mg twice daily 4-hydroxychloroquine 200 mg twice daily 5-hydroxychloroquine 200 mg twice daily 6-Cardiomyopathy with positive gallium scan
